# Patterns of Mandibular Fractures through Human Aggression: A 10-Year Cross-Sectional Cohort Retrospective Study

**DOI:** 10.3390/jcm12124103

**Published:** 2023-06-17

**Authors:** Mircea Rivis, Raluca Iulia Juncar, Abel Emanuel Moca, Rahela Tabita Moca, Mihai Juncar, Paul Andrei Țenț

**Affiliations:** 1 Discipline of Oral Surgery, 2nd Department of Dental Medicine, “Victor Babeș” University of Medicine and Pharmacy, 2 Eftimie Murgu Square, 30041 Timișoara, Romania; rivis.mircea@umft.ro; 2 Department of Dentistry, Faculty of Medicine and Pharmacy, University of Oradea, 10 Piața 1 Decembrie Street, 410073 Oradea, Romania; abelmoca@yahoo.com (A.E.M.); mihaijuncar@gmail.com (M.J.); tent_andrei@yahoo.com (P.A.Ț.); 3 Doctoral School of Biomedical Sciences, University of Oradea, 1 Universității Street, 410087 Oradea, Romania; rahelamoca@gmail.com

**Keywords:** mandibular fractures, interpersonal violence, human aggression

## Abstract

The World Health Organization considers the victims of interpersonal violence to be a medical priority. In order to provide services at the highest level, we aimed to evaluate the patterns of maxillofacial fractures caused by interpersonal violence, in order to treat, counsel and guide these patients. This retrospective study was conducted in 478 patients with mandibular fractures caused by interpersonal violence over 10 years in a university clinic. The most affected were male patients (95.19%), 20–29 years of age (46.86%), under the influence of alcohol (83.26%) and without education (43.9%). The majority of mandibular fractures were displaced (89.3%) and intraorally open (64.0%). The most frequent location was the mandibular angle (34.84%). The most frequent soft tissue lesions were hematomas (45.04%) and abrasions (34.71%), being associated frequently with closed (*p* = 0.945/*p* = 0.237), displaced (*p* = 0.001/*p* = 0.002), single angle fractures (*p* = 0.081/*p* = 0.222). Educating the population and fighting alcohol consumption would decrease the occurrence of mandibular fractures through aggression. Clinical diagnosis should be made, keeping in mind that the severity of associated soft tissue lesions is directly proportional to the pattern and number of underlying fracture lines.

## 1. Introduction

Despite all prevention norms applied worldwide, interpersonal violence (IPV) remains one of the main causes of maxillofacial fractures, having the highest incidence in developed countries [1]. IPV can take many forms, from direct fist hitting or use of blunt objects, to stabbing or gunshots [2].

Due to its prominence in the facial contour, the mandible is the most exposed and fractured bone, secondarily to IPV [3]. Its fracture implies a major morbidity for the patient, because of secondary functional impairment [4]. Mandibular fractures due to IPV have a different clinical picture from those secondary to other etiologies, regarding fracture pattern, degree of bone displacement, and associated soft tissue injuries [5]. Diagnosing and evaluating mandibular fractures caused by IPV in the specialized outpatient services is difficult, due to frequent etiology non-reporting [6,7]. The victim often tries to hide the real cause of trauma because of fear, shame or emotional impact [8]. Classifying a trauma as IPV is extremely important for healthcare financial distribution, the use of medical insurance policies, reimbursement of treatment cost to the victim, and legal procedure faults avoidance [7].

Collaboration with these patients can be frequently difficult, due to the association of trauma with alcohol or drug use, and emotional and psychological implications [1]. An inadequate therapeutic approach in these cases may have major cosmetic and aesthetic implications, which are difficult to control subsequently [1,2,3].

The aim of this study was to evaluate the characteristics, patterns and the type of associated soft tissue injuries of mandibular fractures caused by IPV, as well as to assess the treatment methods applied and their efficiency, by measuring the incidence of postoperative complications. The results of this study will be used for the early clinical diagnosis of mandibular fractures, and also for the determination of whether IPV is an associated factor. We also aimed to identify the main category of affected patients in order to introduce policies for IPV prevention, as well as national specialized programs for the victims’ moral and psychological support.

## 2. Materials and Methods

### 2.1. Ethical Considerations

The study was approved by the Ethics Committee of the University of Oradea, Romania (IRB No. 28765/11.02.2019). All patients included in the study signed, at the time of their admission, an informed consent, by which they agreed to the use of their medical data for scientific research purposes. In the case of patients aged under 18 years, the consent was signed by their parents or legal guardians. This study was approved by the Territorial Ethics Commission, and was therefore performed in accordance with the ethical standards laid down in the 2008 Declaration of Helsinki and its later amendments [9].

### 2.2. Participants and Data Collection

This retrospective study was conducted on a sample of patients admitted and treated over a 10-year period in a university clinic for oral and maxillofacial surgery in north-western Romania.

The PICO (population, intervention, comparison, outcome) question that was used for this study was: “What are the characteristics of patients with mandibular fractures caused by interpersonal violence, and what are the fractures’ patterns, treatment and evolution for these patients?”. The primary outcome was represented by the sample characteristics and fractures’ characteristics, while the secondary outcome was represented by the treatment options and complications.

The study inclusion criteria were: the presence of at least one mandibular fracture line, the written confirmation of IPV etiology by the patient, the presence of imaging investigations in the medical record (X-ray, panoramic radiograph or computed tomography—CT) confirming the clinical diagnosis of fracture and evidencing its topographic location and pattern, treatment of the fracture performed in the clinic hosting this study and postoperative follow-up for at least 6 weeks.

The criteria of exclusion from the study were: patients without any fracture line in the mandible, mandibular fracture of different etiology, patient’s refusal to confirm in writing the IPV etiology, absence of complementary imaging examinations from the patient’s medical record, treatment of the fracture was not performed in the clinic hosting the study (the patient was examined in our outpatient service, but refused admission or chose to be treated in a different center), absence of one or more monitored variables from the medical record, removal of the maxillo-mandibular fixation (MMF) device by the patient sooner than recommended (in the case of patients treated by this method), and impossibility of postoperative follow-up for at least 6 weeks.

Following evaluation of the medical records, 1158 patients were identified to have mandibular fractures caused by IPV during the 10-year period. Of these, 680 patients were excluded from the study for the following reasons: 78 patients had no data referring to the living environment, 52 patients had no data referring to the level of education, 164 patients had no imaging investigations mentioned in the medical record, 110 patients had no data referring to the type of associated soft tissue lesions, 66 patients refused admission to our clinic, and 210 patients did not present to follow-up for at least 6 weeks postoperatively. Thus, the study inclusion criteria were met by 478 patients, having a total number of 726 fracture lines in the mandible.

The following variables were extracted and monitored from the medical records: patients’ gender, age (by life decades), living environment (urban, rural), level of education (no studies—uncompleted primary school, primary school, middle school, high school and university), association of the trauma with alcohol consumption, type of physical aggression (body contact, blunt object, firearm), topographic location of the mandibular fracture, degree of bone involvement, number of fracture lines, association of midface fractures, degree of bone fragment displacement, relationship of the external environment of the fracture, type of associated soft tissue lesions, type of treatment performed, and the incidence, type, and treatment of postoperative complications.

In this study we classified the soft tissue lesions associated with mandibular fractures into: hematoma, laceration, and abrasion. We did not classify post-traumatic edema as an associated soft tissue lesion, this being part of the pathophysiology of the trauma. Thus, post-traumatic edema being present in nearly all facial traumas, fractures or contusions, its classification as a variable is not statistically relevant in this context.

To prevent bias, the first author and one member of the statistical department double-checked the clinical sheets.

The size of this study was achieved due to the 10-year period during which the patients were diagnosed and treated for mandibular fractures in our clinic.

### 2.3. Statistical Analysis

Data were centralized in electronic format using Microsoft Excel software. Descriptive statistics of the cases assessed was performed with a two decimal percent fidelity.

Statistical analysis was carried out with MedCalc Statistical Software version 20.011 (MedCalc Software Ltd., Ostend, Belgium; https://www.medcalc.org, accessed on 13 March 2021). Continuous data were expressed as the mean and standard deviation, while nominal data were expressed as the frequency and percentage. The comparison of the frequencies of a nominal variable between the categories of another nominal variable was performed using the chi-square test. The comparison of a continuous nominal variable between two groups was performed with the T-test for independent variables. A *p* value < 0.05 was considered statistically significant.

We acknowledge that this study was conducted according to the STROBE guidelines.

## 3. Results

### 3.1. Demographics

The most affected age group was 20–29 years, *n* = 224 (46.86%), and the least affected was 70–79 years, *n* = 6 (2.09%). No patient under the age of 10 years was registered. Most of the patients were male, *n* = 455 (95.19%), and the M:F ratio was 19.7:1. The mean age in the case of men was 29.38 years, while for the affected women it was 37.65 years.

The majority of the patients belonged to urban areas, *n* = 263 (55.02%), and had no education, *n* = 216 (45.2%). Most of the patients were under the influence of alcohol at the time of presentation, *n* = 398 (83.26%) (Table 1).

### 3.2. Fractures’ Characteristics

Table 2 shows the different variables investigated with regards to the characteristics of the investigated fractures. Most of the fractures were induced by direct physical contact (*n* = 388, 81.2%). Double mandibular fractures predominated (*n* = 231, 48.3%), and the most frequent location was the mandibular angle (*n* = 253, 34.8%). Most fractures were complete (*n* = 471, 98.5%), displaced (*n* = 427, 89.3%) and open (*n* = 306, 64%). The most frequent soft tissue lesion was hematoma, *n* = 84 (45.04%).

The distribution of the degree of displacement, bone involvement and relationship with the external environment of the fracture depending on the topographic location is found in Table 3. The displacement of the fractured fragments (*p* = 0.256) had a high incidence in the case of patients with angle, body, and parasymphysis fractures, as well as in the case of patients with multiple fracture lines. Further, the patients with multiple, body and parasymphysis fracture lines, had an increased incidence of open fractures (*p* = 0.003).

A high frequency of all associated lesions was identified in the case of patients with multiple mandibular fracture lines (hematoma, *p* = 0.081; laceration, *p* = 0.125; abrasion, *p* = 0.222). In the case of single fractures, a high incidence of hematoma and abrasion can be seen in angle fractures (*p* = 0.081, *p* = 0.222), as well as an increased incidence of laceration in parasymphyseal fractures (*p* = 0.125) (Table 4).

### 3.3. Treatment and Complications

The majority of the patients were treated orthopedically (MMF), *n* = 393 (82.22%) and had a favorable evolution (*n* = 458, 95.8%) (Table 5). Table 6 shows the incidence and the type of complications, depending on the location of the fracture line.

The patients treated exclusively orthopedically (MMF) developed the highest number of complications (*p* = 0.671) (Table 7). In the case of patients who developed osteitis secondarily to MMF, *n* = 12, surgical curettage of the osteitic focus and rigid fixation with miniplates and monocortical screws (ORIF) was performed. In the case of patients who developed osteitis in the osteosynthesis focus, plate and screw removal was performed, with MMF being applied for 6 weeks. All patients had a favorable evolution. In the case of patients with malunion, no surgery was carried out: 2 patients had no functional disorders justifying reintervention, while 3 patients with occlusal disorders refused surgery.

## 4. Discussion

Most of patients with mandibular fractures caused by IPV were male, aged between 20–29 years. Although other authors indicate a predominance of IPV among men, the high male:female ratio (19.7:1) present in our study is uncommon [10,11,12]. Young men, particularly in the third decade of life, are more socially active [5]. They have a more competitive and aggressive behavior than women and, under the influence of alcoholic drinks or drugs, become more liable to engage in conflicts [5,10,11,12]. Acute alcohol poisoning predisposes aggressivity and behavioral alterations in thinking, decision making, and unjustified risk taking [8]. The alcohol-IPV relationship is also highlighted by our results. The higher mean age of women in this study by almost a decade compared to that of men can be explained by the fact that these are most frequently victims of domestic violence (DV), which often manifests after years of cohabitation [13,14,15]. Many authors recommend suspicioning a DV episode in all the cases of women with mandibular fractures through IPV, even if they avoid reporting it [12,13,14,15]. However, physicians should not forget the risk of false accusations of domestic violence to men. Precisely because of these aspects, the approach of these patients must be performed with the utmost accuracy. In contrast to our results, other studies indicate a high incidence of mandibular fractures through IPV among children or teenagers [15]. Although they are, generally, not very frequently reported in children and adolescents, mandibular fractures occurring in these age groups could be caused by domestic violence, and this etiology should be considered [16].

The most affected male patients in this study lived in urban areas, while the majority of women lived in rural areas, however these results have no statistical significance. In contrast, some authors report the highest incidence of mandibular fractures caused by IPV in rural areas, attributing this fact to the low level of education and the poverty of the population [17,18]. These differences can be explained by the type of area that the hospital in which the study was conducted serves [7]. The high incidence of fractures among the population with a low level of education was also found in our results, and is in accordance with the literature [10,14,15,18]. However, the high number of patients without education in our results is rarely found in the literature, and may reveal a major social and educational problem in our geographical area. A low level of education leads to the lack of qualification, unemployment, low or no income, lack of medical insurance, and difficulties in accessing the services necessary to a healthy life [8,18]. All this leads to frustration and envy which, in association with alcohol consumption, can push these patients to violence [10,14,15,18].

The majority of the mandibular fractures in this study occurred through direct physical contact (punches and kicks), followed by hitting with blunt objects, similarly to other studies [2,5,16]. Some authors report a high frequency of single mandibular fractures through direct physical contact, due to the low kinetic energy developed in these cases compared to other etiologies [19,20]. In contrast, in our study, double fractures were predominant. This result suggests an increased violence of injuries through IPV in our geographical area, and also the possibility of multiple blows as part of the same trauma [21]. In our study, no case of mandibular fracture by firearms was registered. Illegality of firearm possession in Romania, as well as the strict criminal legislative norms regarding this aspect, can explain our results. In contrast, in studies conducted in countries where the possession of firearms is permitted and access to them is easy, the incidence of firearm trauma is increased [21,22].

The most frequent location of the fractures registered by us was the mandibular angle, similarly to other studies [1,17,23]. Other authors report the most common location being the parasymphysis [3,24], the body of the mandible [25], the symphysis [10,15], or the subcondylar region [26]. Biomechanically, the mandibular angle is an area of minimum resistance to fracture, due to the presence of the impacted third molar and to the cortical bone thinning at this level [11]. Another aspect that should not be neglected is the anatomical arch shape of the mandible, which often predisposes it to secondary fractures following direct contralateral impact [12,17]. These statements explain our results. No fracture of the coronoid process was registered, similarly to the results of other authors [7].

The majority of the fractures in this study were displaced, similar to results recorded by other authors [3,8,10,23]. Displacement was mainly found in patients with single mandibular angle and body fractures, and in almost all patients with multiple fractures. These results are due to the unequal ratio of insertions of the elevator and depressor muscle groups on the fractured fragments [10,23]. Another factor that should not be neglected is represented by primary displacements through the force and direction of the wounding agent [11,12]. A sufficiently high kinetic energy to induce multiple fracture lines in the mandible will most frequently also cause primary displacements in this category of patients [13,14].

Open fractures were predominant in this study, similarly to other research [3,8,22]. In contrast, other authors indicate a predominance of closed fractures [27,28]. A preponderance of the fracture focus opening was observed in patients with parasymphyseal, body, and multiple fractures, the results being statistically significant. The adherence of the mucoperiosteum to the bone in these anatomical regions predisposes to fracture focus opening through the displacement itself [3,8,22]. This statement is also supported by the high incidence of closed mandibular angle fractures in this study. Despite the great number of displaced fractures registered at this level, the pterygomasseteric sling most frequently prevents fracture focus opening. These statements are supported by other authors [27,28]. In this study, no extraorally open mandibular fractures were identified. The high number of direct bodily contact traumas in this study explains this result [5,19,20]. In contrast, other authors who conducted studies on IPV through firearms indicate a high incidence of extra-orally open fractures [21].

The most frequently associated soft tissue lesion was hematoma, with laceration being the most rarely found [1,11,13]. Contrary to our results, some authors reported an increased incidence of lacerations [2,7,21,26]. We observed an overall increased incidence of soft tissue lesions in patients with multiple fractures. However, these results were not statistically significant. Some authors report that the incidence of lacerations is directly proportional to the number of fracture lines, due to the high kinetic energy that acts with the same intensity in all types of tissue [21,24]. The high incidence of hematoma in our study, even in the case of multiple fractures, suggests the fact that the IPV injuries were less severe. We obtained statistically significant results in the case of associated lesion—fracture pattern correlation. The incidence of soft tissue lesions was directly proportional to the degree of displacement, the opening of the fracture focus, and the degree of bone involvement. Therefore, at clinical examination, the presence of soft tissue lesions is a predictor of a potential underlying mandibular fracture line. These results are supported also by other authors [7,11,24]. These findings are extremely important for the clinician, as the associated lesions and swelling may mask one or more underlying fracture lines. Failure to diagnose mandibular fractures may have severe consequences that are difficult to correct subsequently, the results being sometimes unacceptable for the patient [12]. In Romania, not all specialized centers possess the necessary resources to perform routine CT. In these cases, if the presence of associated soft tissue lesions in a patient represent a CT indication criterion, the patient should be transferred to an adequately equipped specialized center [11].

In this study, the majority of the mandibular fractures were treated by closed reduction and MMF, a result supported by other authors [11,22]. In contrast, the majority of authors treat mandibular fractures solely by ORIF [7,8,12,29], and others by combined (ORIF + MMF) treatment (21). Single ORIF treatment is considered to be the gold standard therapy for mandibular fractures, ensuring rapid healing by direct Haversian remodeling [29]. Further, the absence of MMF secures a healthy marginal periodontium, and facilitates oral hygiene and rapid social reintegration of the patient by maintaining the functionality of the oral cavity [7,8,12,29]. However, there are situations when MMF cannot be avoided. Our results are explained by the duration of the evaluation period of this study (10 years), while in Romania, until 4 years ago, there was no national program ensuring and reimbursing the materials necessary for surgical treatment by ORIF [30]. Thus, under these circumstances, over more than half of the monitored period, the treatment applied to all patients was MMF. Another aspect is that, in severe cases with multiple fracture lines, where the surgeon cannot immediately obtain stable occlusion, some authors recommend ORIF + MMF treatment for at least 2 weeks in order to lower the risk of secondary malocclusions [21]. Subcondylar fractures with considerable displacement should not be overlooked either, where surgical treatment by the transparotid approach involves the risk of facial nerve injury, with transient or even permanent facial paralysis [29]. Under these circumstances, the patient can refuse ORIF treatment, not wanting to take the risk of injury to the facial nerve, or the doctor avoids the surgical approach because of the insufficient mastery of the technique [29,30]. Endoscopic subcondylar fracture reduction by intraoral approach has lately considerably reduced the risk of facial nerve injury, but it is not currently available in Romanian centers [31].

Treatment with MMF developed the greatest number of postoperative complications, results similar to those of other specialized centers [20,21,23,25]. However, the overall rate of complications in this study was low, which may highlight the correct application of this type of treatment [11,22]. The most frequent complications were osteitis and malunion, similarly to other publications [3,7,8,22]. Our results show a high incidence of postoperative complications in the case of displaced and intraorally open fractures. The displacement of bone fragments with the opening of the fracture focus induces direct contamination from the septic oral environment, predisposing these patients to infection and un-union as early as the time of the trauma [3,7,8,22]. Multiple, displaced and open fractures are difficult to treat strictly orthopedically (MMF); incomplete or deficient reduction in these cases increases the rate of complications [30,31]. This was also shown by our results. The fewest complications were found following single ORIF treatment. This result is similar to those of other publications, and reemphasizes the efficacy of perfect open reduction and rigid fixation [7,8,12,29].

This study, however, has limitations due to its retrospective nature. The data from the medical records may be incompletely or incorrectly recorded by clinicians. Further, the possible existence of a much higher number of patients with mandibular fractures caused by IPV during this time period should be taken into consideration, because some patients might have concealed the type of etiology at the time of presentation out of fear or shame. Therefore, selecting only records with complete data might have led to the loss of a great number of cases. It is for these reasons that our results do not have the same impact as those of a prospective randomized controlled study. A prospective study referring to this aspect is required in the future in order to consolidate the results obtained. Another limitation of this study is that we wanted to follow the pattern of fractures secondary to interpersonal aggression in relation to each possible topographic location. Due to the fact that, in some locations, the mandible fractures less frequently, implicitly we had a lower number of patients in that category. In this context, the statistical value may be less representative than in the case of a large number of patients included in all categories.

## 5. Conclusions

The main category of risk for patients with mandibular fractures through IPV is represented by men under the influence of alcohol, aged 20–29 years, living in urban areas and having a low level of education. Most frequently, aggressed patients will present a displaced, intraorally open double mandibular, bicortical fracture. Patients with parasymphysis and mandibular body fractures will most frequently have open fractures, while patients with mandibular angle fractures will have closed fractures. The severity and the incidence of associated soft tissue lesions are directly proportional to the severity of the pattern and the number of underlying fracture lines. Clinical diagnosis, and the need for imaging investigations, should take into consideration this aspect in the case of physically aggressed patients. The most effective treatment method was ORIF, which registered the best success rate and the smallest number of postoperative complications. Educating the population and fighting alcohol consumption by legislative policies, as well as social and psychological assistance of these patients, could lead to a decrease in mandibular fractures caused by IPV.

## Figures and Tables

**Table 1:** Sample characteristics.

Variable	Value
**Age (*n*,%)**	
10–19 years	81 (16.9%)
20–29 years	224 (46.9%)
30–39 years	86 (18%)
40–49 years	46 (9.6%)
50–59 years	25 (5.2%)
60–69 years	10 (2.1%)
70–79 years	6 (1.3%)
**Gender (*n*,%)**	
Male	455 (95.2%)
Female	23 (4.8%)
**Living environment (*n*,%)**	
Rural	215 (45%)
Urban	263 (55%)
**Level of education (*n*,%)**	
No Education	216 (45.2%)
Primary school	26 (5.4%)
Middle school	109 (22.8%)
Highschool	95 (19.9%)
University	32 (6.7%)
**Alcohol consumption (*n*,%)**	
No	80 (16.7%)
Yes	398 (83.3%)

*n*, number; %, percentage.

**Table 2:** Characteristics of the mandibular fractures.

Variable	Value
**Causal agent (*n*,%)**	
Body contact	388 (81.2%)
Blunt object	90 (18.8%)
Firearm	0 (0%)
**Association of midface fractures (*n*,%)**	
No	448 (93.7%)
Yes	30 (6.3%)
**Number of fracture lines (*n*,%)**	
Single	217 (45.4%)
Double	231 (48.3%)
Triple	30 (6.3%)
Comminuted	0 (0%)
**Fracture location (*n*,%)**	
Alveolar Process	2 (0.2%)
Symphysis	22 (3%)
Parasymphysis	120 (16.5%)
Body	168 (23%)
Angle	253 (34.8%)
Ramus	10 (1.4%)
Subcondylar	151 (20.7%)
Condyle Intracapsular	3 (0.4%)
Coronoid Process	0 (0%)
**Degree of bone involvement (*n*,%)**	
Incomplete	7 (1.5%)
Complete	471 (98.5%)
**Displacement (*n*,%)**	
No	51 (10.7%)
Yes	427 (89.3%)
**Relationship with external environment (*n*,%)**	
Closed	172 (36%)
Open	306 (64%)
**Soft tissue lesions (*n*,%)**	
Hematomas	84 (45%)
Lacerations	54 (34.7%)
Abrasions	49 (20.3%)

*n*, number; %, percentage.

**Table 3:** Distribution according to the degree of displacement, bone involvement, and type of fracture, depending on the location of the fracture line.

	Alv. P.	Symphysis	Parasy.	Body	Angle	Ramus	Subcondylar	Condyle I.	Multiple	Total
**Displacement**										
Yes	2	4	16	31	101	2	35	3	233	427
	(0.5%)	(0.9%)	(3.7%)	(7.3%)	(23.7%)	(0.5%)	(8.2%)	(0.7%)	(54.6%)	(100%)
No	0	1	4	7	5	0	6	0	28	51
	(0%)	(2%)	(7.8%)	(13.7%)	(9.8%)	(0%)	(11.8%)	(0%)	(54.9%)	(100%)
Total	2	5	20	38	106	2	41	3	261	478
	(0.4%)	(1%)	(4.2%)	(7.9%)	(22.2%)	(0.4%)	(8.6%)	(0.6%)	(54.6%)	(100%)
Pearson Chi-Square—10.133						*p* = 0.256				
**Bone Involvement**										
Incomplete	0	0	0	0	1	0	0	2	4	7
	(0%)	(0%)	(0%)	(0%)	(14.3%)	(0%)	(0%)	(28.6%)	(57.1%)	(100%)
Complete	2	5	20	38	105	2	41	1	257	471
	(0.4%)	(1%)	(4.2%)	(8.1%)	(22.3%)	(0.4%)	(8.7%)	(0.2%)	(54.6%)	(100%)
Total	2	5	20	38	106	2	41	3	261	478
	(0.4%)	(1%)	(4.2%)	(7.9%)	(22.2%)	(0.4%)	(8.6%)	(0.6%)	(54.6%)	(100%)
Pearson Chi-Square—90.199						*p* = 0.001				
**Type of fracture**										
Closed	2	0	1	1	105	2	41	2	18	172
	(1.2%)	(0%)	(0.6%)	(0.6%)	(61%)	(1.2%)	(23.8%)	(1.2%)	(10.5%)	(100%)
Open	0	5	19	37	1	0	0	1	243	306
	(0%)	(1.6%)	(6.2%)	(12.1%)	(0.3%)	(0%)	(0%)	(0.3%)	(79.4%)	(100%)
Total	2	5	20	38	106	2	41	3	261	478
	(0.4%)	(1%)	(4.2%)	(7.9%)	(22.2%)	(0.4%)	(8.6%)	(0.6%)	(54.6%)	(100%)
Pearson Chi-Square—389.703						*p* = 0.003				

**Table 4:** Distribution of associated soft tissue lesions according to topographic location of the fracture line.

		Hematoma			Laceration			Abrasion		
		No	Yes	Total	No	Yes	Total	No	Yes	Total
**Location**	**Alveolar Process**	1	1	2	2	0	2	1	1	2
		0.3%	1.2%	0.4%	0.5%	0.0%	0.4%	0.2%	1.9%	0.4%
	**Symphysis**	4	1	5	4	1	5	4	1	5
		1.0%	1.2%	1.0%	0.9%	2.0%	1.0%	0.9%	1.9%	1.0%
	**Parasymphysis**	14	6	20	14	6	20	16	4	20
		3.6%	7.1%	4.2%	3.3%	12.2%	4.2%	3.8%	7.4%	4.2%
	**Body**	28	10	38	35	3	38	31	7	38
		7.1%	11.9%	7.9%	8.2%	6.1%	7.9%	7.3%	13.0%	7.9%
	**Angle**	93	13	106	98	8	106	96	10	106
		23.6%	15.5%	22.2%	22.8%	16.3%	22.2%	22.6%	18.5%	22.2%
	**Ramus**	2	0	2	2	0	2	2	0	2
		0.5%	0.0%	0.4%	0.5%	0.0%	0.4%	0.5%	0.0%	0.4%
	**Subcondylar**	32	9	41	38	3	41	39	2	41
		8.1%	10.7%	8.6%	8.9%	6.1%	8.6%	9.2%	3.7%	8.6%
	**Condyle** **Intracapsular**	1	2	3	2	1	3	2	1	3
		0.3%	2.4%	0.6%	0.5%	2.0%	0.6%	0.5%	1.9%	0.6%
	**Multiple**	219	42	261	234	27	261	233	28	261
		55.6%	50.0%	54.6%	54.5%	55.1%	54.6%	55.0%	51.9%	54.6%
**Total**		394	84	478	429	49	478	424	54	478
		100.0%	100.0%	100.0%	100.0%	100.0%	100.0%	100.0%	100.0%	100.0%
		Pearson Chi-Square 14.028		*p* = 0.081	Pearson Chi-Square 12.647		*p* = 0.125	Pearson Chi-Square 10.650		*p* = 0.222

**Table 5:** Treatment options and evolution.

Variable	Value
**Treatment (*n*,%)**	
Orthopedic (MMF)	393 (82.2%)
Combined (ORIF + MMF)	46 (9.6%)
Surgically (ORIF)	39 (8.2%)
**Evolution (*n*,%)**	
Favorable	458 (95.8%)
Unfavorable	20 (4.2%)

*n*, number; %, percentage.

**Table 6:** Distribution of postoperative complications, depending on the location of the fracture.

		Complications			
		None	Osteitis	Malunion	Total
**Location**	**Alveolar Process**	2	0	0	2
		0.4%	0.0%	0.0%	0.4%
	**Symphysis**	5	0	0	5
		1.1%	0.0%	0.0%	1.1%
	**Parasymphysis**	20	0	0	20
		4.4%	0.0%	0.0%	4.2%
	**Body**	36	1	1	38
		7.9%	7.7%	20.0%	8.0%
	**Angle**	102	6	0	106
		22.3%	30.8%	0.0%	22.3%
	**Ramus**	2	0	0	2
		0.4%	0.0%	0.0%	0.4%
	**Subcondylar**	41	0	0	41
		9.0%	0.0%	0.0%	8.6%
	**Condyle Intracapsular**	3	0	0	3
		0.7%	0.0%	0.0%	0.6%
	**Multiple**	247	10	4	261
		53.9%	61.5%	80.0%	54.4%
	**Total**	458	15	5	478
		(100%)	(100%)	(100%)	(100%)
Pearson Chi-Square—6.061			*p* = 0.003		

**Table 7:** Distribution of postoperative complications, depending on the treatment method applied.

		Complications			
		None	Osteitis	Malunion	Total
**Type of treatment**	**Closed MMF**	377	12	4	393
		82.3%	92.3%	80.0%	82.6%
	**ORIF**	39	1	0	40
		8.5%	0.0%	0.0%	8.2%
	**Compound**	42	2	1	45
		9.2%	7.7%	20.0%	9.2%
	**Total**	458	15	5	478
		(100%)	(100%)	(100%)	(100%)
Pearson Chi-Square—2.352			*p* = 0.671		

## Data Availability

The data presented in this study are available on request from the corresponding authors. The data are not publicly available due to privacy reasons.
